# Au Clusters Treat Rheumatoid Arthritis with Uniquely Reversing Cartilage/Bone Destruction

**DOI:** 10.1002/advs.201801671

**Published:** 2019-02-15

**Authors:** Fuping Gao, Qing Yuan, Pengju Cai, Liang Gao, Lina Zhao, Meiqing Liu, Yawen Yao, Zhifang Chai, Xueyun Gao

**Affiliations:** ^1^ CAS Key Laboratory for the Biological Effects of Nanomaterials and Nanosafety Institute of High Energy Physics Chinese Academy of Sciences Beijing 100049 China; ^2^ Department of Chemistry and Chemical Engineering Beijing University of Technology Beijing 100124 China; ^3^ State Key Laboratory of Radiation Medicine and Protection Soochow University Suzhou, Jiangsu 215123 China

**Keywords:** Au clusters, bone destruction, inflammation, rheumatoid arthritis

## Abstract

Super‐small nanoclusters may intrinsically trigger specific molecular pathway for disease treatment in vitro/vivo. To prove the hypothesis the super‐small nanoclusters, e.g., Au clusters, are directly used to treat rheumatoid arthritis (RA) in vitro/vivo. RA is a chronic autoimmune disease that is characterized by the inflammation of joints and the unreversible destruction of the cartilage/bone. Au clusters significantly suppress lipopolysaccharide (LPS)‐induced proinflammatory mediator production in the murine macrophage cell line by inhibiting the signaling pathways that regulate the major proinflammatory mediator genes. In preclinical rat RA studies, Au clusters strongly prevent type II collagen‐induced rat RA without systemic side effects. Compared with the clinical first‐line anchored anti‐RA drug, methotrexate, Au clusters equally inhibit inflammation in vivo. Type II collagen‐induced rat RA is characterized with the destruction of cartilage/bone; treatment with Au clusters reverses the destruction of cartilage/bone to its normal state. This is because Au clusters directly inhibit receptor activator of nuclear factor‐κB ligand (RANKL)‐induced osteoclast differentiation and function through the downregulation of osteoclast‐specific genetic marker expression. However the methotrexate almost has no positive effect for this key issue in rat RA therapy. These data prove that the super‐small nanoclusters, e.g., Au clusters, could be a novel candidate nanodrug for RA treatment.

## 1 Introduction

Rheumatoid arthritis (RA) is principally characterized by synovial inflammation of the joints and the unreversible destruction of cartilage/bone.^1^ Patients with RA suffer from cartilage/bone destruction induced disability, systemic complications, and early death, which result in socioeconomic costs. Because the pathogenesis of RA has not been fully elucidated, the current therapeutic strategy is to use disease‐modifying antirheumatic drugs (DMARDs) to reduce inflammation, relieve pain, suppress disease activity, and slow cartilage/bone destruction.^2^ Methotrexate (MTX) is the first‐line anchored DMARD for the treatment of RA in clinical practice.^3^ However, long term use of MTX may cause severe systemic complications and it is almost with no positive effects for cartilage/bone destruction in RA treatment.^4^ Newer treatments using biologic therapies, such as biopharmaceuticals that inhibit tumor‐necrosis factor (TNF),^5^ have shown notable achievement in the treatment of RA. Nonetheless, there are still unmet medical needs as a lack of patient and/or disease responsiveness, the resistance to long‐term treatment, concerns regarding high costs, and increased infection risks of these biologic agents in the treatment of RA.^6^ Therefore, the discovery of more effective and safer drug for RA therapy is desired in the world.

Molecular clusters of Au have special physicochemical and biological properties due to their precise molecular structure,^7^ that differ substantially from those of the corresponding compounds containing gold and Au nanoparticles with larger particle sizes. Owing to their ultrasmall size, good biocompatibility, high stability, robust preparation, and intrinsic biological activity, metal nanoclusters exhibit great potentials for application in biomedicine.^8^ Although compounds containing gold in the Au(I) state, such as sodium aurothiomalate, sodium aurothiosulfate, aurothioglucose, and auranofin are able to arrest the progression of RA, in up to one‐third of the patients treatment has to be stopped, they are rarely used in clinical practice because of high toxicity and adverse side effects.^9^ Compared with monovalent gold compounds, Au clusters are more highly biocompatible. Hence in this report, we sought to develop Au clusters as a new nanotherapeutic drug for the treatment of RA in preclinical classic RA rat model. This novel nanodrug definition is very different from traditional nanodrug concept where chemical/biological drug molecules loaded in nanoparticles and the nanoparticles just work as delivery system, it is chemical/biological drug molecules rather nanoparticles treat disease in vitro/vivo. In this study, gold clusters as a new nanodrugs, have a direct biological effect, rather than using a drug carrier. We further figure out how Au clusters directly trigger specific molecular pathways to suppress the inflammation of joints and recover the destruction of cartilage/bone to its normal state in vitro/vivo.

## 2 Results

### 2.1 Characterization of Au Clusters

**Figure 1:**
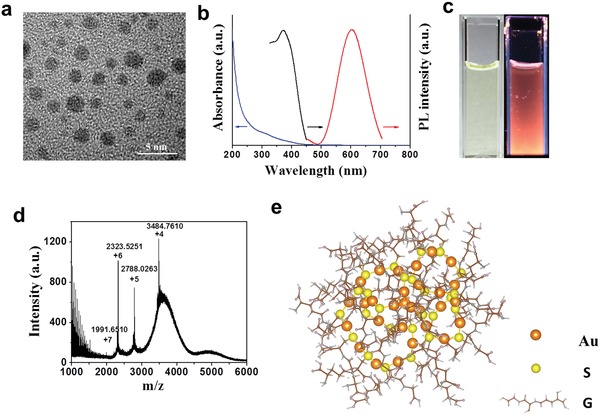
Characterizations of the Au clusters, Au_29_SG_27_, synthesized using glutathione (GSH = γ‐Glu‐Cys‐Gly) as the template. a) High‐resolution transmission electron microscope (HRTEM) image of the Au clusters. b) UV–vis absorption (blue lines), photoemission (red lines, λ_ex_ = 365 nm), and photoexcitation (black lines, λ_em_ = 610 nm) spectra of the Au clusters at room temperature. c) Digital photos of the Au clusters in deionized water under visible (left) and UV (right) light. d) Electrospray ionization mass spectrometry (ESI‐MS) of the Au clusters. ESI mass spectra of the clusters display a series of multiple charged cations originating from the addition of formic acid during testing. The molecular weight can be determined from deconvoluted spectra by combinations of Au atoms and GS ligands. The 4^+^ (*m*/*z* 3484.7610), 5^+^ (*m*/*z* 2788.0263), 6^+^ (*m*/*z* 2323.5251), and 7^+^ (*m*/*z* 1991.6510) peaks can be deconvoluted into the corresponding mass of an uncharged cluster (*m*/*z* 13 935). Such a cluster could be assigned as Au_29_SG_27_ with a molecular weight of 13 983 Da, which may lose a carboxyl group during the ionization process. e) Schematic diagram of the Au_29_SG_27_ molecular structure by density functional theory. The Au atoms and S atoms are in orange and yellow, respectively. G indicates the glutathione skeleton without S atoms and H atoms.

In this study, Au clusters were synthesized using a natural tripeptide, glutathione (GSH = γ‐Glu‐Cys‐Gly) as the thiolate ligand to bind the produced Au clusters, the purified Au clusters were designated GA. As small naturally occurring peptides, GSH could be a good surface ligand for Au clusters due to ensuring a small hydrodynamic diameter, providing good interface with the biological system and improving their in vivo pharmacokinetics. The Au clusters were super‐small and well dispersed, as shown in high‐resolution transmission electron microscope (HRTEM) images (**Figure** **1**a). The hydrodynamic sizes of the Au clusters were 2.11 ± 0.5 nm which was determined using dynamic light scattering (DLS) (Figure S1, Supporting Information). The zeta potential of the Au clusters was −29 mV in deionized water and −23.8 mV in pH 7.4 phosphate buffered saline (PBS) attributed to the net charge of GS shell. Once the Au clusters formed, they presented luminescence characteristics. Au clusters showed absorption peaks at 330 nm in the UV–vis region (Figure 1b, blue lines). The optical excitation/emission properties of the Au clusters were examined, and excitation (black lines) and emission peaks (red lines) at 372 and 604 nm, respectively, were observed (Figure 1b). The Au clusters were a light yellow in solution under visible light, and they exhibited red fluorescence under UV light at 365 nm (Figure 1c). The emission peak of the Au clusters hardly changed after being incubated in deionized water, physiological saline, fetal bovine serum (FBS), high glucose Dulbecco's modified Eagle's medium (DMEM) medium, and pH 7.4 PBS for 24 h at room temperature (Figure S2, Supporting Information). The appearance and fluorescence characteristics of the Au clusters did not change significantly after 10 days at 4 °C (Figure S3, Supporting Information). These results show that the synthesized Au clusters have higher stability. The molecular formula of the Au clusters was Au_29_SG_27_, as determined by electrospray ionization mass spectrometry (ESI‐MS) based on the reported literature (Figure 1d).^10^ The detailed molecular composition and structure of the Au clusters, which were obtained using density of functional theory (DFT) calculations (Molecular modeling of Au_29_SG_27_ in Section S3, Supporting Information), are shown in Figure 1e.

### 2.2 The Anti‐Inflammatory Effect of the Au Clusters in vitro

**Figure 2:**
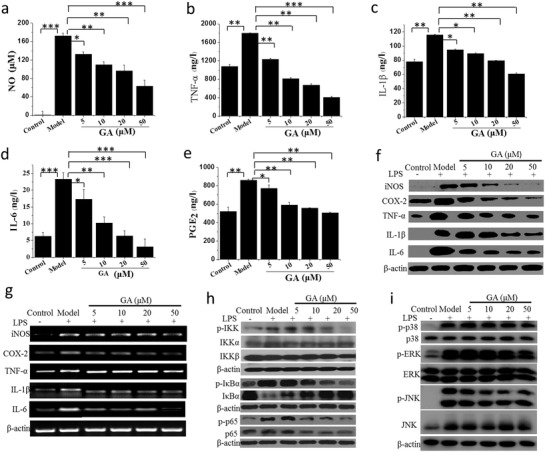
Au clusters synthesized with glutathione as the template (Au_29_SG_27_, designated GA) inhibit the inflammatory response of lipopolysaccharide (LPS )‐stimulated RAW 264.7 cells. a–e) Concentration‐dependent inhibition of inflammatory mediators NO a), TNF‐α b), IL‐1β c), IL‐6 d), and PGE_2_ e) in LPS‐stimulated RAW 264.7 cells treated with different concentrations of Au clusters. NO released in the cell supernatants was measured using Griess reagent and an ELISA plate reader. TNF‐α, IL‐1β, IL‐6, and PGE_2_ were measured by ELISA analysis. Data (*n* = 6, mean ± SD) are representative of at least two independent experiments. f) Concentration‐dependent inhibition of the i‐NOS, COX‐2, TNF‐α, IL‐1β, and IL‐6 protein levels in LPS‐stimulated RAW 264.7 cells measured by western blot analysis. β‐actin was used as an internal control. Images are representative of two experiments. g) Concentration‐dependent inhibition of the i‐NOS, COX‐2, TNF‐α, IL‐1β, and IL‐6 mRNA levels in LPS‐stimulated RAW 264.7 cells analyzed by real‐time RT‐PCR using specific primers. β‐actin was used as an internal control. Images are representative of two experiments. h) Inhibitory effect of different concentration of Au clusters on IKK, IκBα, and p65 phosphorylation in LPS‐stimulated RAW 264.7 cells analyzed by western blotting. β‐actin was used as an internal control. Images are representative of two experiments. i) Inhibitory effect of different concentrations of Au clusters on MAPK signaling pathways in LPS‐stimulated RAW 264.7 cells analyzed by western blotting. β‐actin was used as an internal control. Images are representative of two experiments. Data are presented as the mean ± SD; ^*^ *p* < 0.05, ^**^ *p* < 0.01, ^***^ *p* < 0.001.

The murine macrophage cell line RAW 264.7 was used in this investigation as it is widely employed as a model cell line in RA studies. Prior to examining the anti‐inflammatory effect of Au clusters in vitro, we first determined their cytotoxicity in RAW 264.7 cells. No significant cytotoxic effects were observed after incubating the RAW 264.7 cells with concentrations of Au clusters ranging from 0.1 × 10^−6^ to 100 × 10^−6^ m for 24–48 h (Figure S4, Supporting Information). However, monovalent gold compound, auranofin reduced RAW 264.7 cells viability to 50% even at 5 × 10^−6^ m concentration of Au contained after 24 h of incubation (Figure S5, Supporting Information). The results indicate that Au clusters as a new gold preparation have higher biocompatibility and lower toxicity than monovalent gold compound. Next, we examined the effect of Au clusters on lipopolysaccharide (LPS)‐induced inflammation in RAW 264.7 cells. Macrophages play an important role in the pathogenesis of RA, and macrophage‐derived cytokines, such as TNF‐α, interleukin‐1β (IL‐1β), and interleukin‐6 (IL‐6), are relatively abundant in the rheumatoid synovium. As shown in **Figure** **2**, when various concentrations of Au clusters (5 × 10^−6^, 10 × 10^−6^, 20 × 10^−6^, or 50 × 10^−6^ m in Au dose) were added to the culture media at the time of cell stimulation (24 h), the Au clusters suppressed LPS‐induced secretion as well as the production of proinflammatory mediators including nitric oxide (NO) (Figure 2a), TNF‐α (Figure 2b), IL‐1β (Figure 2c), IL‐6 (Figure 2d), and prostaglandins (PGE_2_) (Figure 2e) by RAW 264.7 cells in a dose‐dependent manner. These proinflammatory mediators interact with each other and collectively play an important role in initiating and perpetuating inflammatory and bone destructive processes in the rheumatoid joint. TNF‐α and IL‐1β regulate the expression of other cytokines and proinflammatory mediators, such as cyclooxygenase‐2 (COX‐2) and inducible nitric oxide synthase (i‐NOS), which control the production of PGE_2_ and NO, respectively.^11^ PGE_2_ promotes inflammatory angiogenesis in the synovium by inducing vascular endothelial growth factor expression,^12^ and both PGE_2_ and NO participate in destructive mechanisms in the rheumatoid joint.^11^ We next examined the intracellular levels of i‐NOS, inflammatory biomarkers, COX‐2, and proinflammatory cytokines via western blot analysis in LPS‐stimulated cells, again showing that Au clusters decreased the cellular levels of i‐NOS, PGE_2_, TNF‐α, IL‐1β, and IL‐6 induced by LPS in a dose‐dependent manner (Figure 2f). Au clusters also dose‐dependently suppressed the mRNA levels of i‐NOS, PGE_2_, TNF‐α, IL‐1β, and IL‐6 in LPS‐stimulated cells, as determined by RT‐PCR analysis (Figure 2g). These findings suggest that Au clusters play a significant role in suppressing inflammatory responses through LPS stimulation. In immunity and inflammation, the transcription factors nuclear factor (NF)‐κB and mitogen‐activated protein kinase (MAPK) are part of major signaling pathways that regulate the major proinflammatory mediator genes.^13^ In unstimulated cells, the two‐subunit p50/p65 heterodimer of NF‐κB is sequestered in the cytoplasm in its inactivated state through interaction with inhibitory protein IκB. Inflammatory signals induce the activation of the IκB kinase (IKK) complex, which causes ubiquitination, phosphorylation, and subsequent degradation of IκB proteins, and the released NF‐κB enters the nucleus (especially p65 subunit) to induce the expression of specific target genes.^14^ As shown in Figure 2h, Au cluster treatment blocked IKK activation, which in turn suppressed IκB degradation and p65 activation. LPS stimulation induces the phosphorylation and activation of three MAPKs: JNK, ERK, and p38 MAPK.^15^ We found that Au clusters also dose‐dependently inhibited the phosphorylation of three types of MAPKs, especially the phosphorylation of JNK. These results provide a potential explanation for the global decrease in inflammatory mediator levels in the LPS‐stimulated macrophages observed in Figure 2a–e.

**Figure 3:**
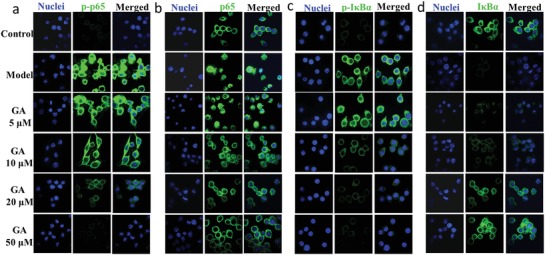
Indirect immunofluorescence assay and confocal fluorescence images of IκBα degradation and p65 nuclear localization. a,b) Concentration‐dependent inhibition of phosphor‐p65 (p‐p65) and p65 nuclear localization by Au clusters in LPS‐stimulated RAW 264.7 cells visualized with confocal microscopy after immunofluorescence staining with p‐p65 antibody a) or p65 antibody b) (green). Cells were stained with DAPI to visualize the nuclei (column labeled nuclei). c,d) Concentration‐dependent inhibition of phosphorylation and degradation of IκBα by Au clusters in LPS‐stimulated RAW 264.7 cells visualized with confocal microscopy after immunofluorescence staining with p‐IκBα antibody c) or IκBα antibody d) (green). Cells were stained with DAPI to visualize the nuclei (column labeled nuclei). The images are representative of two experiments.

Degradation of IκB and the subcellular localization of p65 were also visualized with confocal microscopy after immunofluorescence staining with an IκB antibody, a phosphorylated IκB antibody, a p65 antibody, or a phosphorylated p65 antibody. Confocal images showed that IκB and p65 were normally retained in the cytoplasmic compartment (**Figure** **3**b,d, control panel). LPS stimulation strongly induced the phosphorylation and degradation of IκB and the nuclear accumulation of p65 in RAW 264.7 cells (Figure 3a–d, model panel). Au clusters dose‐dependently inhibited the phosphorylation and degradation of IκB and the phosphorylation and translocation of p65 in LPS‐stimulated RAW 264.7 cells (Figure 3a–d, GA panel). These results were consistent with western blot analyses of IκB and p65 (Figure 2h).

### 2.3 Au Clusters Ameliorate Collagen‐Induced Arthritis (CIA) in Rats

**Figure 4:**
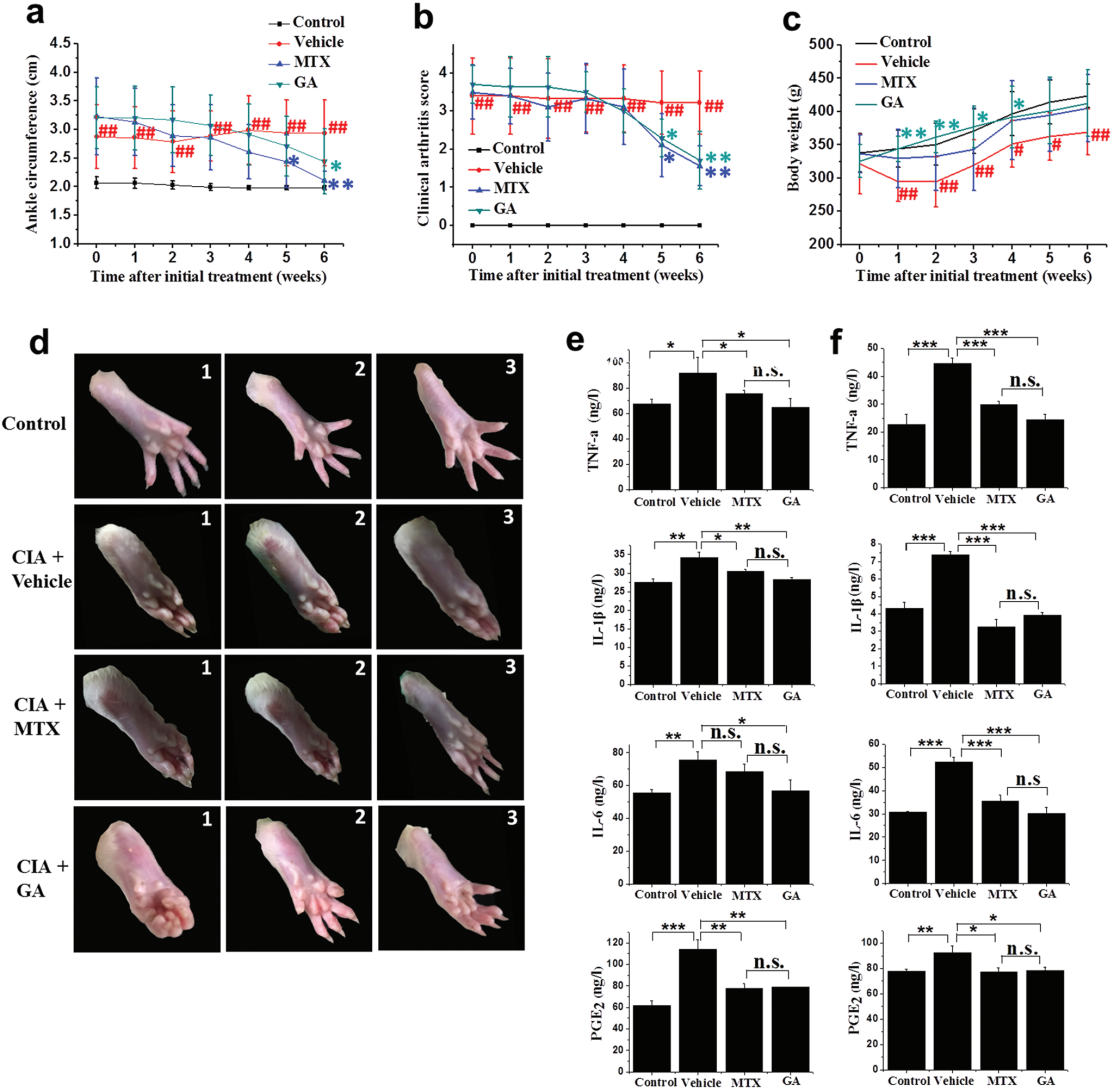
Au clusters inhibit inflammatory responses in rat CIA. a–c) Progression of ankle circumference a), clinical arthritis score b), and body weight c) in CIA rats (*n* = 10) over 6 weeks after starting treatment with vehicle (buffer), MTX, and Au clusters. Nonimmunized normal rats treated with vehicle were used as a control group. d) Representative photographs of CIA rats treated with vehicle, MTX, and Au clusters and nonimmunized normal rats at the 1) initial, 2) middle, and 3) end timepoints of drug administration. e,f) The levels of proinflammatory cytokines (TNF‐a, IL‐1β, and IL‐6) and PGE_2_ at the end of the study (day 42) in the serum e) and articular tissue f) of CIA rats treated with vehicle (buffer), MTX, and Au clusters. The cytokine and PGE_2_ levels of nonimmunized rats in serum and articular tissue were used as controls. Data are presented as the mean ± SD; ^#^ *p* < 0.05 and ^##^ *p* < 0.01 versus nonimmunized control rats for ankle circumference, clinical arthritis score, and body weight; ^*^ *p* < 0.05, ^**^ *p* < 0.01, and ^***^ *p* < 0.001 versus CIA rats treated with vehicle. For proinflammatory cytokines and PGE_2_ at the end of the study in serum (e) and articular tissue (f), ^*^ *p* < 0.05, ^**^ *p* < 0.01, ^***^ *p* < 0.001, and n.s., not significant.

Based on the encouraging in vitro effects of the Au clusters via the inhibition of inflammation described above, we next considered whether Au clusters might also exert anti‐inflammatory and immunomodulatory in vivo efficacy in an experimental arthritis animal model. The anti‐inflammatory effects of Au clusters were evaluated in the rat CIA model,^16^ which is the classic CIA model widely accepted in RA studies and has compelling key features of the inflammatory response observed in human RA.^17^ MTX was selected as a positive control drug for in vivo anti‐inflammatory response because of its precise anti‐inflammatory and immunomodulatory effects and because it is frequently used as a first‐line anchored drug for the treatment of RA.[[qv: 3b]] Prior to the formal evaluation of in vivo anti‐inflammatory effects, we first tested the acute toxicity of Au clusters in rats. The results showed that the lethal dose 50 (LD_50_) of Au clusters was 288.9 ± 13 mg kg^−1^ bw after intraperitoneal injection, this dose was nearly 39 times more than the LD_50_ of Au(I) compound auranofin and 57 times more than the LD_50_ of the traditional MTX (5 mg kg^−1^ bw, intraperitoneal injection), indicating its lower toxicity. As the intraperitoneal injection of Au clusters resulted in higher bioavailability than oral administration (Figure S6, Supporting Information), we used intraperitoneal administration for in vivo anti‐inflammatory experiments. The rats induced with CIA were treated at disease onset (day 22 post primary collagen immunization) with Au clusters (injected intraperitoneally, 5 mg of Au kg^−1^, once a day) or MTX (administered orally, 0.5 mg kg^−1^, twice a week).^18^ The Au cluster dosing was based on a pilot study that investigated a series of doses in a CIA rat model to obtain the optimal dose of Au clusters. The rats in the nonimmunized normal control group and the CIA model control group were injected intraperitoneally with an equal volume of saline. After 1 h of intraperitoneal injection, the blood concentration of Au clusters peaked. Half‐life was 7.5 h. After six weeks of treatment, the kidney showed the highest distribution of Au clusters, indicating that the Au clusters were mainly excreted through the kidneys (Figure S7, Supporting Information). As shown in **Figure** **4**a,b, after treatment for 4–5 weeks, the Au clusters had an obvious anti‐RA effect similar to that of MTX, as evaluated by ankle circumference and clinical arthritis score. Although MTX treatment showed a slight improvement in ankle circumference over time compared with the Au cluster treatment, there was no statistical significance between the MTX‐treated group and the Au cluster‐treated group (Figure 4a). After the CIA model was established, the rats suffered severe body weight loss due to inflammation when compared to the normal rats, and this loss in body weight was sustained up to 6 weeks. After the administration of Au clusters and MTX, the CIA rats exhibited significant body weight gain, especially the Au cluster‐treated rats, and their body weight gain was comparable to that of the normal rats during the 6 weeks of treatment (Figure 4c). These data suggest that the administration of Au clusters prevents inflammatory body weight loss in CIA rats. In addition, after 6 weeks of Au cluster treatment, all hematology and biochemical indexes were within normal ranges (Tables S1 and S2, Supporting Information). Major organ tissues including the heart, liver, lung, spleen, and kidney showed no notable histopathological abnormalities or lesions in any of the groups (Figure S8, Supporting Information). These results demonstrated that the Au clusters did not cause unexpected side effects and could be used as novel nanodrug for the treatment of RA. Representative photos of the joints and claws of rats in the four groups at the initial, middle, and end time points of drug administration are shown in Figure 4d, providing visible evidence of the anti‐inflammatory effects of Au clusters when compared with the saline‐treated CIA model rats.

We also examined the effect of Au clusters on biochemical parameters in CIA rats by measuring serum cytokine (TNF‐α, IL‐1β, and IL‐6) and PGE_2_ levels after drug treatment for 6 weeks. Compared to the baseline serum cytokine and PGE_2_ levels in nonimmunized normal rats, serum TNF‐α, IL‐1β, IL‐6, and PGE_2_ levels were significantly (*p* < 0.05) increased in CIA rats after immunization (Figure 4e). Compared to the saline‐injected CIA rats, the Au cluster‐treated CIA rats had significantly decreased serum TNF‐a, IL‐1β, IL‐6, and PGE_2_ levels (Figure 4e). Similar to the serum cytokine and PGE_2_ levels, the articular tissue TNF‐α, IL‐1β, IL‐6, and PGE_2_ levels were also significantly (*p* < 0.05) decreased in the paws of Au cluster‐treated CIA rats in contrast to those in the saline‐injected CIA rats (Figure 4f). The reduction in cytokine and PGE_2_ levels due to treatment with Au clusters in CIA rats was comparable to that of MTX treatment, whether in serum or tissue.

**Figure 5:**
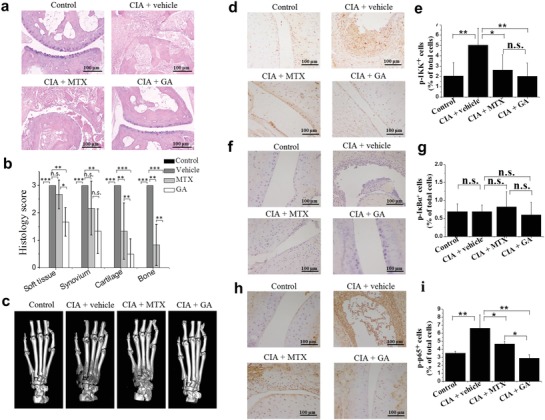
The therapeutic effects of Au clusters in the rat CIA model. a) Representative histopathological images (*n* = 6 per group) of joint sections from nonimmunized rats and CIA rats treated with vehicle, MTX, and Au clusters on day 42. (Hematoxylin and Eosin staining; Scale bar = 100 µm). b) Histological scores of inflammation and cartilage and bone erosion in CIA rats treated with vehicle, MTX, and Au clusters on day 42. The joint sections of nonimmunized rats were used as controls. c) Representative microCT images (*n* = 6 per group) of metatarsal bone and ankle articulations in CIA rats treated with vehicle, MTX, and Au clusters on day 42. MicroCT images of metatarsal bone and ankle articulations of nonimmunized rats were used as controls. d–i) Representative images of immunohistochemistry (IHC) of phosphor‐IKK, phosphor‐IκBα, and phosphor‐p65 antigenicity (d,f,h) (3 mice per group) and percentages of p‐IKK^+^, p‐IκBα^+^, and p‐ p65^+^ cells (e,g,i) in joint tissues from nonimmunized rats and CIA rats treated with vehicle, MTX, and Au clusters on day 42 (Scale bar = 100 µm). Synovial cells and inflammatory cells in the articular cavity were stained brown to indicate phosphor‐IKK and phosphor‐p65 antigenicity. Data are presented as the mean ± SD; ^*^ *p* < 0.05, ^**^ *p* < 0.01, ^***^ *p* < 0.001; n.s., not significant.

We further examined the therapeutic effect of Au clusters through a histopathological analysis and 3D microcomputed tomography (microCT) imaging of the joints of rats at the conclusion of the study (after 6 weeks of drugs treatment). RA rats treated with vehicle showed severe inflammation and cartilage and bone destruction (**Figure** **5**a). The interface between cartilage and bone was hard to distinguish due to inflammatory cell infiltration, synovial hyperplasia, pannus formation, and cartilage and bone destruction. Treatment with Au clusters abrogated the characteristic signs of CIA‐induced chronic inflammation. A clear interface between cartilage and bone could be seen, and it was comparable to tissue sections of the nonimmunized normal control group rats (Figure 5a). MTX treatment showed only a partial reduction in signs of inflammation. The mean pathological score of soft tissue, synovial tissue, cartilage, and bone obtained from histopathological analyses of the joints of five rats further demonstrated that treatment with Au clusters significantly (*p* < 0.01) reduced pathological scores compared with those of RA rats treated with vehicle. For cartilage and bone, Au clusters showed a stronger (*p* < 0.05) anti‐inflammatory effect than MTX (Figure 5b). In agreement, microCT imaging showed that the extent of bone erosion in the hind paws of representative rats was markedly disparate in the different treatment groups (Figure 5c). The bone erosion and joint deformity observed in CIA rats were completely abrogated by treatment with Au clusters (Figure 5c and Figure S8, Supporting Information). However, MTX treatment did not markedly improve bone erosion in CIA rats (Figure 5c and Figure S9, Supporting Information).

Immunohistochemical analysis revealed increases in activated IKK complex and p65 phosphorylation in the joint synovial cells and inflammatory cells of CIA rats (Figure 5d,e; h,i). Au clusters treatment led to marked reduction in activated IKK protein levels and p65 phosphorylation, which were comparable to the levels of phosphor‐IKK and phosphor‐p65 in the joint tissues of nonimmunized normal rats. MTX treatment only partially reduced the levels of phosphor‐p65 in the joint tissues of CIA rats (Figure 5h,i). We did not detect apparent expression of phosphorylated IκBα in the joint tissue of CIA rats, which was likely due to the degradation of IκB proteins (Figure 5f,g). These results are consistent with the in vitro experiments and provide a potential explanation for the global decrease in inflammatory cytokine levels observed in blood and articular tissue, as shown in Figure 4,f.

### 2.4 The Inhibitory Effect of Au Clusters on the Formation and Differentiation of Osteoclasts (OCs)

Both histopathological analysis and microCT imaging demonstrated that Au clusters reduced severe bone destruction better than MTX (Figure 5a–c). Changes in the fine bone structure determined from microCT coronal imaging and quantification of bone mineral density (BMD) further showed that the severe loss of bone density in CIA rats was almost completely recovered by treatment with Au clusters (Figure S9, Supporting Information). However, MTX treatment had almost no effect on bone density when compared with that of CIA rats treated with vehicle. The BMD value of the Au cluster‐treated group was almost two times that of the vehicle‐treated group and the MTX‐treated group (Figure S9, Supporting Information). The formation and differentiation of OCs is directly involved in bone resorption. In the pathological state of rheumatoid immune and inflammatory conditions, increased OC formation induces excessive bone resorption.^19^ Therefore, we next considered whether Au clusters have a direct inhibitory effect on the formation and differentiation of OCs. To confirm this hypothesis, we returned to an in vitro system. We examined the effect of Au clusters on OC differentiation by incubating bone marrow‐derived monocytes (BMMs) in OC formation induction media containing the osteoclastogenic cytokine receptor activator of nuclear factor‐κB ligand (RANKL, 50 ng mL^−1^) and macrophage‐colony stimulating factor (M‐CSF, 30 ng mL^−1^).

**Figure 6:**
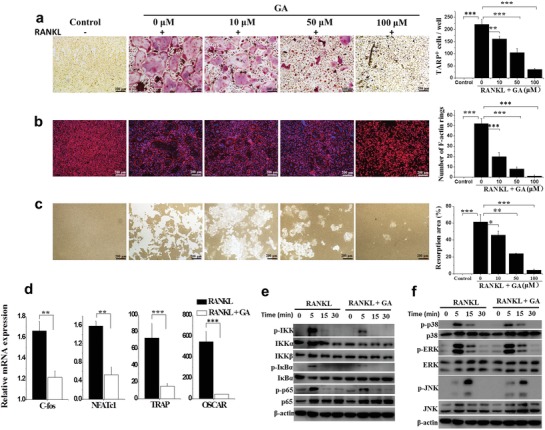
Au clusters directly inhibit RANKL‐induced OC formation and differentiation. a) Representative TRAP staining images (*n* = 9) (left) and the number of TRAP‐positive multinucleated cells after 4 days of differentiation (right), demonstrating the effect of different concentrations of Au clusters on OC formation. Scale bar = 100 µm. b) Representative phalloidin staining images (*n* = 9) from the actin ring formation assays; cell nuclei were stained blue using DAPI (left). Scale bar = 100 µm. The number of intact F‐actin rings (right). c) Representative images (*n* = 9) of bone resorption pits generated by mature OCs after 6 days and the percentage resorption area (right). Scale bar = 100 µm. M‐CSF (30 ng mL^−1^) and RANKL (50 ng mL^−1^) were present in all of the OC formation and differentiation assays. Control cells cultured without RANKL and Au clusters were used as negative controls. d) The relative mRNA expression levels of c‐fos, NFATc1, TRAP, and OSCAR in the presence of RANKL with or without Au clusters, relative to GAPDH. e) western blot analysis of protein expression levels of phosphorylated IKK, IκBα, p65, p38, ERK, and JNK in BMMs pretreated with or without Au clusters for 4 h prior to RANKL stimulation (50 ng mL^−1^) for the indicated times. β‐actin was used as an internal control. Images are representative of two experiments. Data are presented as the mean ± SD; *p < 0.05, ^**^ *p* < 0.01, ^***^ *p* < 0.001.

Prior to testing the effects of Au clusters on OC differentiation, we first examined the cytotoxicity of Au clusters in BMMs using a CCK‐8 kit (Figure S10, Supporting Information). We found that Au clusters were not cytotoxic to BMMs, even at concentrations of 500 × 10^−6^ m. OC marker tartrate‐resistant acid phosphatase (TRAP) staining was utilized to detect the effect of Au clusters on OC differentiation. As shown in **Figure** **6**a, BMMs efficiently differentiated into TRAP^+^ multinuclear cells (MNCs) in the absence of Au clusters, while the formation of TRAP^+^ MNCs was decreased in a dose‐dependent manner in the presence of Au clusters. The actin ring is a unique cytoskeletal structure of OCs that allows them to resorb bone. We detected actin ring formation in BMMs incubated in OC induction media by immunofluorescence analysis in the absence or presence of Au clusters. Supplementation with Au clusters dose‐dependently decreased the actin ring formation stimulated by RANKL. Both the size and number of F‐actin rings decreased after Au cluster treatment (Figure 6b). Likewise, Au clusters inhibited OC‐mediated resorption pit formation in bone slices in a dose‐dependent manner. Quantitative analysis of bone slices showed that the percentage area of resorption pits gradually decreased with increasing Au cluster concentrations (Figure 6c). Consistently, Au cluster treatment also significantly inhibited the number of OC precursors in the articular tissues of CIA rats, which was indicated by the number of RANK‐positive stained OCs. In the immunohistochemical analysis of RANK antigenicity in joint tissues, we found that the RANK expression level in the joint tissue of CIA rats treated with Au clusters was as low as that in normal rats (Figure S11, Supporting Information). We also examined the effect of Au clusters on the specific genetic markers of OC differentiation, including c‐Fos, nuclear factor of activated T cells 1 (NFATc1), TRAP, and OC‐associated receptor (OSCAR) in RANKL‐treated BMMs via real‐time PCR. c‐Fos and NFATc1 are the most important OC‐specific transcriptional regulators involved in OC differentiation.^20^ RANKL stimulation induced the upregulation of c‐Fos, NFATc1, and the osteoclastogenic genes TRAP and OSCAR. Treatment with Au clusters led to downregulation of the mRNA expression levels of c‐Fos, NFATc1, TRAP, and OSCAR that were stimulated by RANKL in BMMs (Figure 6d). We next examined the RANKL‐induced activation/inactivation levels of signaling pathways including MAPKs (p38, ERK, and JNK) and NF‐κB (IKK, IκBα, and p65) in BMMs, which regulate the process of OC differentiation.[[qv: 19a,21]] Au cluster treatment reduced the expression levels of p‐IKK, p‐IκBα, and p‐p65 or p‐p38, p‐ERK, and p‐JNK at 5 or 15 min, respectively (Figure 6e,f). Collectively, these results demonstrated that Au clusters notably prevented excessive bone erosion in RA joints.

## 3 Discussion

Because the aetiology and pathogenesis of RA remain poorly understood, effective therapies with limited side effects are still lacking. The current treatment strategy is to inhibit the inflammatory process and prevent bone damage.[[qv: 2a]] Although monovalent gold drugs have been used for the treatment of RA patients for more than 70 years, poor efficacy or adverse side effects often result in the discontinuation of treatment.^9^ Gold drugs now appear to be rarely used clinically. The monovalent state of gold drugs, Au(I), when not tightly bound to ligands, will spontaneously dismutate to generate zero valent gold (Au^0^) and trivalent gold (Au^+3^) in vivo.^22^ Au^0^ is the active drug, and Au^+3^ may be responsible for the toxicity observed during the treatment of RA with monovalent gold drugs.^9^, ^23^ This supposition inspired us to design and develop new gold drugs that combine maximum therapy efficacy with minimal side effects. In this work, a single Au cluster was composed of 29 gold atoms in its core with a shell comprising 27 GSH molecules bound to the Au_29_ cluster via Au—S bonds (Figure 1d,e and Molecular modeling of Au_29_SG_27_ in Section S3, Supporting Information). As expected, the Au clusters suppressed the secretion and production of inflammatory mediators by LPS‐stimulated RAW 264.7 macrophages in a dose‐dependent manner. LPS stimulation induced the activation of downstream signals, including the IκB/NF‐κB and MAPK pathways, through its binding to toll‐like receptor 4 (TLR‐4).^13^ NF‐κB and MAPKs are part of major signaling pathways that control the transcription of inflammatory mediators.^24^ Our results indicated that LPS‐induced NF‐κB activation, phosphorylation and degradation of IκBα, and activation of the IKK complex were significantly inhibited by Au clusters (Figures 2h and 3). Au clusters also significantly suppressed phosphorylation of the MAPKs (p38, ERK, and JNK) (Figure 2i). These findings demonstrate that Au clusters achieve anti‐inflammatory effects by regulating the signaling pathways driven by NF‐κB and MAPK activation.

Given the complexity of the in vivo environment and the pathophysiology of RA, we first determined whether Au clusters could produce an effective anti‐inflammatory effect in an animal model of RA. In this study, the CIA rat model was used for studying the in vivo anti‐inflammatory effect of Au clusters. The CIA is classic preclinical model and it reproduces many pathogenic mechanisms similar to those of human RA, such as pannus formation, synovial hyperplasia, cellular infiltration, and erosion of cartilage and bone in joints.[[qv: 14b]] The results showed that the Au clusters had an effect similar to that of MTX in reducing ankle circumference and clinical arthritis score in the CIA rats (Figure 4a,b). However, microCT imaging and histopathological analysis showed that Au clusters more potently protected bone destruction than MTX (Figure 5a–c and Figure S8, Supporting Information). Bone tissue remodeling depends on the dynamic balance between bone formation by osteoblast activation and bone resorption by OC activation. In the inflammatory environment, the cytokines produced by macrophages of the synovial tissue indirectly mediate skeletal destruction during RA.^25^ In the current study, Au cluster treatment reduced the serum and joint tissue levels of TNF‐α, IL‐1β, and IL‐6 in arthritic rats in a manner that was almost similar to the effect of MTX (Figure 4e,f). RANKL, which is a key regulator of osteoclastogenesis and OC differentiation, binds to its receptor, RANK, and induces a series of intracellular signaling pathways that drive mononuclear phagocyte precursors to differentiate into multinucleated OCs.^26^ Given the in vivo anti‐inflammatory findings regarding the protection of bone destruction (Figure 5), we speculated whether Au clusters have a direct inhibitory effect on RANKL‐induced OC differentiation and function. The in vitro results showed that treatment with Au clusters decreased the number of OCs and impaired the structure (F‐actin rings) and bone resorptive activity of these cells in a dose‐dependent manner (Figure 6a–c). We also found that OC‐specific genes, including TRAP and OSCAR, were downregulated. The mRNA expression levels of two key transcriptional factors (c‐fos and NFATc1) were also downregulated (Figure 6d), these factors regulate the expression of OC‐specific genes and control OC differentiation.[[qv: 20b,27]] The RANKL tune two major intracellular signaling pathways, e.g., NF‐κB and MAPKs (p38, ERK, and JNK), which further regulate the processes of OC differentiation and maturation as well as the expression of osteoclastogenic genes.^28^ The results clearly showed that Au clusters effectively inhibited the RANKL induced NF‐κB and MAPK pathways, including phosphorylation and degradation of IκBα, phosphorylation of p65 and IKK, and phosphorylation of p38, ERK, and JNK at predefined time points (Figure 6e,f). Taken together, these data demonstrated that the Au clusters directly inhibited OC formation and function by suppressing the RANKL induced NF‐κB and MAPK signaling pathways and their downstream transcription factors, c‐fos, NFATc1, and osteoclastogenic genes.

## 4 Conclusions

In summary, the present study demonstrates that Au clusters with a specific molecular structure, i.e., Au_29_SG_27_, play profound anti‐inflammatory roles by inhibiting inflammatory cytokines and almost recover destructive cartilage/bone to its normal states for rat RA, which was more pronounced than that of the first‐line clinical anchored anti‐RA drug MTX. Au clusters suppress cartilage/bone destruction via directly inhibiting the formation and differentiation of OC induced by RANKL through the suppression of the NF‐κB and MAPK signaling pathways. Compared with the Au(I) compound auranofin and traditional MTX, Au clusters had a far higher LD_50_. The treatment of CIA rats with Au clusters did not result in any undesirable side effects. After later detailed study of their mechanism of action, Au clusters may have great potential to become an attractive novel nanodrug for RA and other chronic inflammatory and autoimmune disorders.

## 5 Experimental Section

*Synthesis and Characterization of Glutathione‐Protected Au Clusters*: Glutathione‐protected Au clusters were fabricated according to the previously reported method with modification.^29^ Freshly prepared aqueous solutions of glutathione (GSH = γ‐Glu‐Cys‐Gly) (Sigma‐Aldrich, USA) (30 × 10^−3^ m, 100 mL) and hydrogen tetrachloroaurate trihydrate (HAuCl_4_·3H_2_O) (Sigma‐Aldrich, USA) (20 × 10^−3^ m, 100 mL) were mixed under gentle stirring (500 rpm) at 25 °C for 10 min. Subsequently, the reaction mixture was heated to 70 °C under gentle stirring (500 rpm) for 12 h, after which it was kept at room temperature in the dark for an additional 12 h. A solution of strongly orange‐emitting Au clusters was obtained. The clusters were purified by adding 30 mL of ethanol to 10 mL of the as‐synthesized clusters. The solution was mixed well and then centrifuged at 10 000 rpm for 15 min. Under such conditions, the supernatant containing the free GSH and gold ions was discarded, and the clusters precipitated out of the solution were washed three times using an ultrapure water and ethanol mixture (1:3 volume ratio). The purified clusters were dried under vacuum and redispersed in ultrapure water with the assistance of sodium hydroxide. For further purification, the clusters were purified through an ultrafiltration tube (Millipore, MWCO: 3 KDa) to remove free ions. UV–vis absorption and photoluminescence (PL) spectra of synthesized clusters were recorded using a Shimadzu UV‐1800 photospectrometer (Japan) and a Shimadzu RF‐5301 fluorescence spectrophotometer (Japan), respectively. The molecular composition of the synthesized clusters was analyzed by ESI‐MS. A Bruker microTOF‐Q ESI time‐of‐flight system operating in the positive ion mode was used at a sample injection rate of 25 µL min^−1^ and a capillary voltage of 4 kV with the nebulizer at 1.5 bar, dry gas at 4 L min^−1^ at 160 °C, and an *m*/*z* of 1000–6000. For the stability study, the synthesized Au clusters were incubated in deionized water, physiological saline, FBS, DMEM medium, and pH 7.4 PBS for 24 h at room temperature. The fluorescence intensity of Au clusters was detected. The appearance of precipitation and fluorescence characteristics under UV light at 365 nm were observed by visual inspection.

*Cell Culture*: Mouse macrophage RAW 264.7 cells were obtained from the American Type Culture Collection (ATCC). The cells were cultured in Dulbecco's modified Eagle's medium‐F12 (DMEM/F12 1:1) (GibcoTM) supplemented with 10% inactivated FBS, 100 U mL^−1^ penicillin, and 100 µg mL^−1^ streptomycin in 5% CO_2_ and 95% air at 37 °C. The cells were seeded on 24‐well plates (5 × 10^5^ cells mL^−1^) and treated with 1 µg mL^−1^ LPS (Sigma‐Aldrich, USA) and various concentrations of Au clusters in serum‐free medium.

*The Induction of OC Formation in vitro*: Osteoclastogenesis was induced by primary cultured rat BMMs. Monocytes were isolated from the femoral and tibial bone marrow of 2‐week‐old SD rats^30^ and then cultured in α‐MEM supplemented with 10% FBS, 100 U mL^−1^ penicillin, 100 µg mL^−1^ streptomycin, and 30 ng mL^−1^ mouse M‐CSF (R&D Systems) at 37 °C in a humidified 5% CO_2_ atmosphere. During cell culture, nonadherent cells were removed by changing the medium. After reaching ≈90% confluence, the cells were digested with trypsin and inoculated into culture plates for further experiments. For induction of OC differentiation, the cells were cultured in media containing 50 ng mL^−1^ mouse RANKL (R&D Systems) and 30 ng mL^−1^ M‐CSF without or with different concentrations of Au clusters for the indicated days. The culture medium containing different concentrations of Au clusters was changed every two days.

*Cell Viability Assay*: The RAW 264.7 cells or BMMs were inoculated into 96‐well culture plates at 5 × 10^3^ cells per well. After incubation overnight, the culture medium was then replaced with different concentrations of Au clusters or monovalent gold compound, auranofin. The cells were incubated at 37 °C in a 5% CO_2_ atmosphere for 24–48 h. The cell viability was investigated using a cell counting kit (CCK‐8) (Dojindo Molecular Technologies Inc., Japan) assay.

*In vitro OC Formation and Resorption Assay*: OC formation was evaluated by TRAP staining and actin ring formation assays. After osteoclastogenic induction for 4 days in the presence of different doses of Au clusters (10 × 10^−6^, 50 × 10^−6^, and 100 × 10^−6^ m), the BMMs were washed with PBS once and fixed in 3.7% paraformaldehyde for 15 min at room temperature, followed by permeabilization with 0.1% Triton X‐100 for 10 min. Fixed cells were stained with a TRAP staining kit (387A‐1KT, Sigma‐Aldrich, USA) for 1 h at 37 °C in the dark following the manufacturer's instructions. TRAP‐positive multinucleated (nuclei > 3) cells were counted as OCs using a light microscope (IX71; Olympus).

For the actin ring formation assay, BMMs were seeded onto glass‐bottom dishes (35 mm, MatTek Corporation) at a density of 2 × 10^4^ cells per dish and were incubated in inducing medium for 4 days in the presence of different doses of Au clusters (10 × 10^−6^, 50 × 10^−6^, and 100 × 10^−6^ m) to form actin rings. Then, the cells were washed twice with PBS and fixed in 3.7% paraformaldehyde for 20 min, followed by permeabilization with 0.1% Triton X‐100 for 15 min at room temperature. Actin rings were stained by rhodamine‐conjugated phalloidin (Cytoskeleton, Inc., Denver, CO) after washing with PBS, and cell nuclei were stained with DAPI (Invitrogen) for 10 min. The formation of actin rings in each sample was visualized by confocal laser scanning microscopy (Nikon Ti‐E imaging system, Tokyo, Japan).

Resorption activity was evaluated by OC‐mediated pit formation. BMMs were cultured in Corning Osteo Assay Surface 24‐well plates coated with calcium phosphate substrate at a density of 2 × 10^4^ cells/well. The medium was removed after 24 h of incubation, and inducing medium containing different doses of Au clusters (10 × 10^−6^, 50 × 10^−6^, and 100 × 10^−6^ m) was added. The culture medium was refreshed every two days. After ≈6 days, the cells were removed with a 10% sodium hypochlorite solution and rinsed three times with water. The bone resorption pits were observed and photographed using a light microscope. ImageJ (1.46r) software was used to quantify the percentage of resorbed bone surface area.

*Measurement of NO Release*: RAW 264.7 cells were seeded into 96‐well plates (1 × 10^5^ cells/well) and treated with or without LPS (1 µg mL^−1^) and different concentrations of Au clusters (5, 10, 20 and 50 µmol L^−1^). After incubation for 24 h at 37 °C, 100 µL of culture supernatant was mixed with an equivalent volume of Griess reagent (0.1% *N*‐[1‐naphthyl]‐ethylenediamine and 1% sulfanilamide in 5% phosphoric acid) (Beyotime Biotechnology, China) and incubated at room temperature for 10 min. The absorbance at 540 nm was measured using a microplate absorbance reader (Bio‐Rad Laboratories Inc.), and a series of known concentrations of sodium nitrite was used to construct a standard curve.

*Enzyme‐Linked Immunosorbent Assay (ELISA)*: The concentrations of TNF‐α, IL‐1β, IL‐6, and PGE_2_ in the culture supernatants and the serum and joint tissues lysate supernatants from rats were determined by ELISAs using TNF‐α, IL‐1β, IL‐6, and PGE_2_ ELISA kits (Shanghai Haling Biological Technology Co., Ltd., China) according to the manufacturer's instructions.

*Western Blot Analysis*: RAW 264.7 cells were seeded into 6‐well plates at a density of 2 × 10^6^ cells/well. After incubation without or with LPS (1 µg mL^−1^) and different concentration of Au nanoclusters for 24 h, the cells were collected and lysed with RIPA buffer (50 mmol L^−1^ Tris‐HCl, pH 7.4, 150 mmol L^−1^ NaCl, 1% Triton X‐100, 1% sodium deoxycholate, 0.1% SDS, 1 mmol L^−1^ sodium orthovanadate, 50 mmol L^−1^ NaF, and 1 mmol L^−1^ ethylenediaminetetraacetic acid) along with protease inhibitor (Roche Molecular Biochemicals). The lysate was centrifuged at 13 000 rpm for 10 min, and the supernatant was stored for subsequent analysis. The concentration of protein was determined using a microplate spectrophotometer (SpectraMax M4, Molecular Devices, USA) at a wavelength of 595 nm. An equal quantity of protein (50 µg) was separated by 10% SDS‐PAGE and transferred to a polyvinylidene fluoride (PVDF) membrane (0.45 µm, Millipore, USA). After blocking, the membrane was incubated with specific antibodies for i‐NOS (Cell Signaling Technologies, 13120, 1:1000), COX‐2 (Cell Signaling Technologies, 12282, 1:1000), IL‐1β (Cell Signaling Technologies, 12703, 1:1000), IL‐6 (Cell Signaling Technologies, 12912, 1:1000), TNF‐α (Cell Signaling Technologies, 11948, 1:1000), phosphor‐p65 (Cell Signaling Technologies, 3033, 1:1000), p65 (Cell Signaling Technologies, 3034, 1:1000), phosphor‐IκBα (Cell Signaling Technologies, 2859, 1:1000), IκBα (Cell Signaling Technologies, 4812, 1:1000), IKKα (Cell Signaling Technologies, 2682, 1:1000), IKKβ (Cell Signaling Technologies, 8943, 1:1000), phosphor‐IKKα/β (Cell Signaling Technologies, 2697, 1:1000), phosphor‐p38 (Thr180/Tyr182) (Cell Signaling Technology, 9211, 1:200), p38 (Cell Signaling Technology, 9212, 1:1000), phosphor‐Erk (Thr202/Tyr204) (Cell Signaling Technology, 9101, 1:1000), Erk (Cell Signaling Technology, 4695, 1:1000), phosphor‐JNK (Thr183/Tyr185) (Cell Signaling Technology, 9251, 1:1000), JNK (Cell Signaling Technology, 9258, 1:1000), and actin (Cell Signaling Technologies, 4970, 1:5000), followed by incubation with an appropriate secondary antibody conjugated to horseradish peroxidase (Beyotime Biotechnology, China). To detect protein in BMMs, the cells were stimulated with 30 ng mL^−1^ M‐CSF and 50 ng mL^−1^ RANKL for 5, 15, and 30 min along with 100 µmol L^−1^ Au clusters; the cells were then lysed and subjected to western blotting as described above using specific antibodies for MAPK and NF‐κB signaling pathway analysis.

*Reverse‐Transcriptase PCR and Quantitative Real‐Time PCR (qPCR)*: Total RNA was extracted using an RNeasy Plus Mini Kit (Qiagen NV, Venlo, the Netherlands). First, 1 µg of purified total RNA was reverse transcribed into complementary DNA using an Oligo(dT)15 Primer (Promega Corporation, Fitchburg, WI, USA) and M‐MLV Reverse Transcriptase (Promega) following the manufacturer's instructions. The concentrations of mRNA were quantified by absorption at 260 nm. Equal amounts of mRNA were used to perform PCR with the corresponding primers, and β‐actin was used as an internal control. The amplification products were visualized by agarose gel electrophoresis. The oligonucleotide primers used were as follows: i‐NOS, sense, 5′‐AGCTCCTCCCAGGACCACAC‐3′, and antisense, 5′‐ACGCTGAGTACCTCATTGGC‐3′; COX‐2, sense, 5′‐ACGGAGAGAGTTCATCCCTGACCC‐3′, and antisense, 5′‐TGACTGTGGGGGGATACACCTCTC‐3′; IL‐1β, sense, 5′‐CAGGATGAGGACATGAGCACC‐3′, and antisense, 5′‐CTCTGCAGACTCAAACTCCAC‐3′; TNF‐α, sense, 5′‐CCTGTAGCCCACGTCGTAGC‐3′, and antisense, 5′‐TTGACCTCAGCGCTGAGTTG‐3′; IL‐6, sense, 5′‐GTACTCCAGAAGACCAGAGG‐3′, and antisense, 5′‐TGCTGGTGACAACCACGGCC‐3′; and β‐actin, sense, 5′‐GTGGGCCGCCCTAGGCACCAG‐3′, and antisense, 5′‐GGAGGAAGAGGATGCGGCAGT‐3′.

qPCR was performed with the iQ5 Multicolor Real‐Time PCR Detection System (Bio‐Rad Laboratories, Inc., Hercules, CA, USA) according to the manufacturer's recommendations. Total RNA was extracted using an RNeasy Plus Mini Kit (Qiagen NV, Venlo, the Netherlands). Purified RNA was reverse transcribed into complementary DNA by using an Oligo(dT)15 Primer (Promega Corporation, Fitchburg, WI, USA) and M‐MLV Reverse Transcriptase (Promega) following the manufacturer's instructions. qPCR was performed with the iQ SYBR Green Supermix (Bio‐Rad) via the standard protocol. Sequences of the primers were as follows: c‐fos, sense, 5′‐GGATTTGACTGGAGGTCTGC‐3′, and antisense, 5′‐TTGCTGATGCTCTTGACTGG‐3′; NFATc1, sense, 5′‐CTCGAAAGACAGCACTGGAGCAT‐3′, and antisense, 5′‐CGGCTGCCTTCCGTCTCATAG‐3′; TRAP, sense, 5′‐CTGGAGTGCACGATGCCAGCGACA‐3′, and antisense, 5′‐TCCGTGCTCGGCGATGGACCAGA‐3′; OSCAR, sense, 5′‐TCTGCCCCCTATGTGCTATC‐3′, and antisense, 5′‐CTCCTGCTGTGCCAATCAC‐3′; and glyceraldehyde 3‐phosphate dehydrogenase (GAPDH), sense, 5′‐CATGGCCTTCCGTGTTCCTACCC‐3′, and antisense, 5′‐CCTCAGTGTAGCCCAAGATGCCCT‐3′. The amplification factor was calculated by the comparative threshold cycle method. The ratios of gene expression fold changes were calculated using GAPDH as a control.

*Immunofluorescence*: RAW 264.7 cells were seeded onto glass‐bottom dishes (35 mm, MatTek Corporation) and treated with or without LPS (1 µg mL^−1^) and/or 5–50 µmol L^−1^ of Au clusters. After 24 h, the cells were fixed with 4% paraformaldehyde for 15 min, permeabilized with 0.5% Triton X‐100 for 20 min, and blocked with 5% BSA in PBS at room temperature for 1 h. Then, the cells were incubated with the primary antibodies for phosphor‐p65 (1:100), p65 (1:100), phosphor‐IκBα (1:100), and IκBα (1:100) at 37 °C for 1 h, followed by a 1 h incubation with fluorescein isothiocyanate (FITC)‐conjugated goat‐antirabbit IgG (Beyotime, China). The cell nuclei were stained with 10 µg mL^−1^ DAPI (Invitrogen) for 10 min. After being washed three times with PBS, the cells were observed and imaged by confocal laser scanning microscopy (Nikon Ti‐e microscope, CLSM) with excitation at wavelengths of 405 and 488 nm.

*CIA and Treatment Protocols*: All animal care and experiments were conducted in compliance with the requirements of the National Act on the use of experimental animals (China) and were approved by the Institutional Animal Care and Ethic Committee at the Chinese Academy of Sciences (Approved No. SYXK (jing) 2014‐0023). An immune emulsion was prepared by dissolving bovine type II collagen (CII) (Chondrex, Inc.) in 0.1 × 10^−3^ m acetic acid at 4 °C overnight and emulsifying with an equal volume of incomplete Freund's adjuvant (Chondrex, Inc). Male 5‐ to 6‐week‐old Wistar rats (Beijing Vital River Laboratory Animal Technology Co., Ltd.) were intradermally injected at the base of the tail with 0.2 mL of the emulsion containing 400 µg of CII. On day 7 after the primary immunization, the rats were boosted intradermally with 200 µg of CII. Rats were closely monitored for arthritis disease severity and progression by ankle circumference, clinical arthritis score, and body weight. The clinical arthritis score was assessed by grading each paw from 0 to 4 according to the extent of erythema and swelling: score 0 = no erythema or swelling; score 1 = slight erythema or swelling of one of the toes or fingers; score 2 = erythema and swelling of more than one toe or finger; score 3 = erythema and swelling of the ankle or wrist; score 4 = complete erythema and swelling of toes or fingers and ankle or wrist.^31^ Each limb was graded, and a mean score was given for each animal.

At 14 days after the secondary CII immunization, and only when the clinical scores reached 3–4, the rats were randomly assigned to the following groups, each consisting of 10 animals: Group I, saline‐injected normal control; Group II, saline‐injected CIA control; Group III, arthritic rats orally administered MTX (Shanghai Xinyi Pharmaceutical Co., Ltd, China; Batch number: 036151102) as reference (0.5 mg kg^−1^, twice a week); and Group IV, arthritic rats injected (i.p.) with Au clusters in saline (5 mg of Au kg^−1^ day^−1^). Treatment was initiated from day 22 and continued up to day 64 after primary immunization. On day 64 after arthritis induction, blood was collected from all the groups of animals for hematological and blood biochemical evaluation as well as for the measurement of cytokine levels in serum specimens. Rats were euthanized by excess CO_2_ inhalation, and ankle joint tissues were isolated from the hind paws of rats and homogenized with T‐PER reagent containing 1 × cocktail protease inhibitor for the detection of tissue cytokines. The major organs were excised and fixed in 10% formalin for histopathological examination.

*MicroCT Analyses and Histopathological Analysis*: The hind limb of each rat was harvested after sacrifice and imaged with 3D microcomputed tomography (microCT, Siemens Inveon MM Gantry CT, Germany) at a voltage of 70 kV and an electric current of 400 µA. The exposure time was 800 ms, and the scan area was 26.42 mm × 26.42 mm × 30 mm around the metatarsal bone articulations. 3D analysis and BMD were analyzed using microCT software.

For histopathological analysis, hind paws were fixed in 10% formalin after removal of the skin and were then decalcified in 5% formic acid, embedded in paraffin, microtomically sectioned into 5 µm slices, and stained with hematoxylin and eosin.

*Immunohistochemistry*: The hind paws were treated as above for histopathological analysis, except they were decalcified with 10% EDTA instead of 5% formic acid. The 5 µm tissue sections were deparaffinized, rehydrated, and rinsed in PBS. To inactivate endogenous peroxide, 3% H_2_O_2_ was applied. Antigens in the tissue sections were repaired by heating for 20 min in sodium citrate buffer at 95 °C in a microwave oven, followed by cooling for 20 min at room temperature. The sections were blocked with 5% goat serum; incubated with primary antibodies against phosphor‐p65 (Abcam, ab86299, 1:70), phosphor‐IκBα (Geme Tex, GTX32224, 1:30), phosphor‐IKKα/β (Geme Tex, GTX52310, 1:50), and RANK (Bioss, bs‐2695R, 1:50) at 37 °C for 70 min; and then incubated for 30 min with HRP‐conjugated secondary antibody (Beyotime, China) at 37 °C. After rinsing extensively, the sections were incubated with a diaminobenzidine (DAB) solution for 3 min at room temperature and were then counterstained with hematoxylin. The images were observed and analyzed using an Olympus CX31 light microscope.

*Statistical Analyses*: Sample size was determined according to preliminary data and observed effect sizes. Data are represented as the mean ± SD Data were previously tested with homogeneity of variance using Levene's test. Statistical significance of overall differences between multiple groups was determined by one‐way analysis of variance. If the test was significant, pairwise comparisons were performed using the Student's *t*‐test. *p* < 0.05 was considered statistically significant.

## Conflict of Interest

The authors declare no conflict of interest.

## Supporting information

Supplementary
