# Supplementary material for: Au Clusters Treat Rheumatoid Arthritis with Uniquely Reversing Cartilage/Bone Destruction
Source: Adv Sci (Weinh). 2019 Feb 15;6(7):1801671. doi: 10.1002/advs.201801671 (PMC6446600; doi:10.1002/advs.201801671)
Supplement: Supplementary file 1 — Supplementary [file ADVS-6-1801671-s001.pdf]

Copyright WILEY-VCH Verlag GmbH & Co. KGaA, 69469 Weinheim, Germany, 2019.

ADVANCED SCIENCE
Open Access

Supporting Information

for *Adv. Sci.,* DOI: 10.1002/advs.201801671

Au Clusters Treat Rheumatoid Arthritis with Uniquely Reversing Cartilage/Bone Destruction

*Fuping Gao, Qing Yuan, Pengju Cai, Liang Gao, Lina Zhao, Meiqing Liu, Yawen Yao, Zhifang Chai, and Xueyun Gao**

Copyright WILEY-VCH Verlag GmbH & Co. KGaA, 69469 Weinheim, Germany, 2019.

Supporting Information

Au Clusters Treat Rheumatoid Arthritis with Uniquely Reversing Cartilage/Bone Destruction

Fuping Gao, Qing Yuan, Pengju Cai, Liang Gao, Lina Zhao, Meiqing Liu, Yawen Yao, Zhifang Chai, and Xueyun Gao*

1. **Supplementary Figures**

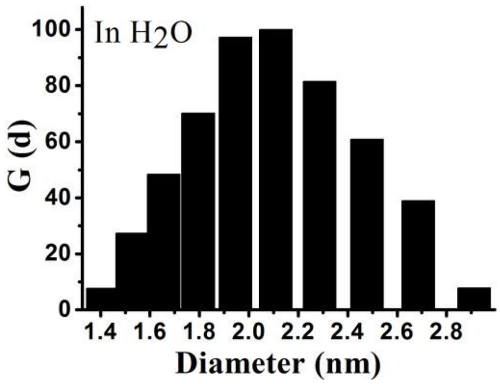

Figure S1. Size distribution of Au clusters by dynamic light scattering (DLS).

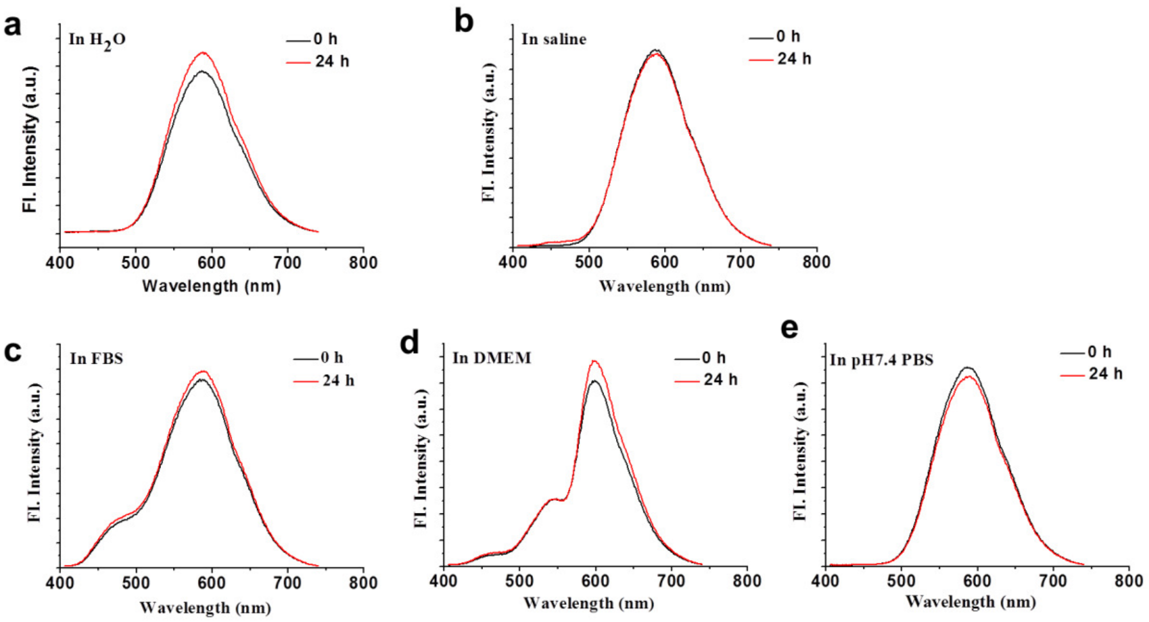

Figure S2. The fluorescence emission spectra of Au clusters dispersed in deionized water, physiological saline, FBS, DMEM medium, and pH 7.4 PBS at freshly prepared and after 24 hours at room temperature. The emission peak of the Au clusters hardly changed after incubation.

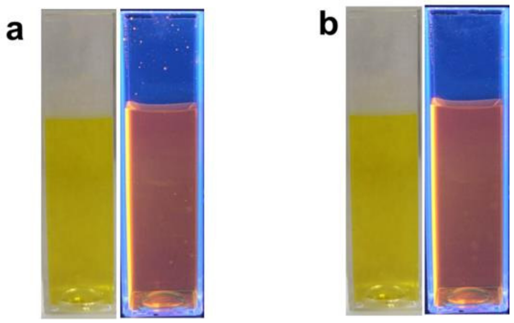

Figure S3. The photographs of Au clusters in deionized water under visible (left) and UV (right) light at freshly prepared (a) and after 10 days (b) at 4 ^{o}C.

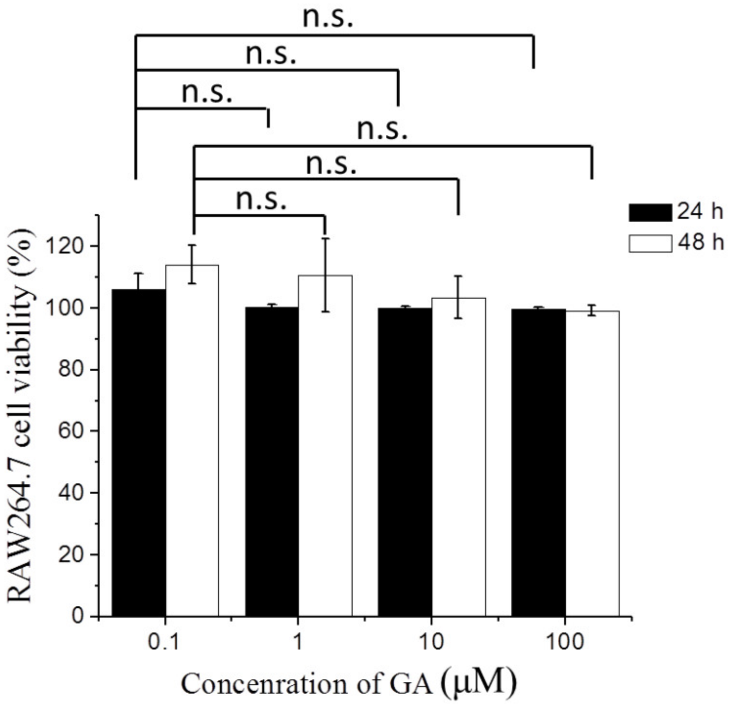

Figure S4. Viability of RAW 264.7 cells treated with different concentrations of Au clusters for 24 h or 48 h at 37 °C. Data are presented as the mean ± s.d. (n = 8); n.s., not significant.

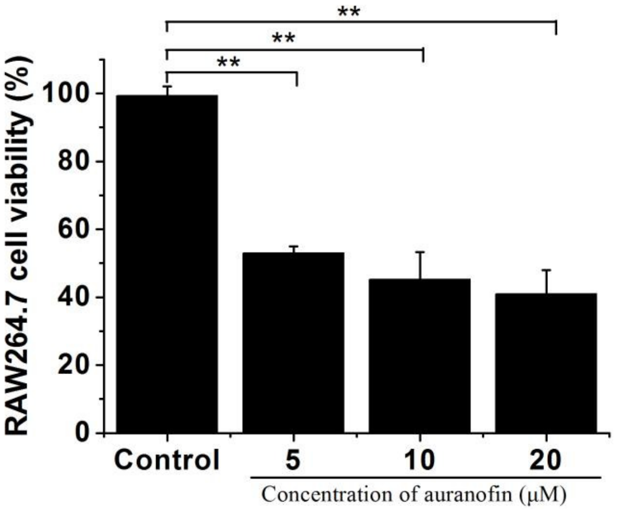

Figure S5. Viability of RAW 264.7 cells treated with different concentrations of monovalent gold compound, auranofin for 24 h at 37 °C. Data are presented as the mean ± s.d. (n = 8); **P ＜ 0.01.

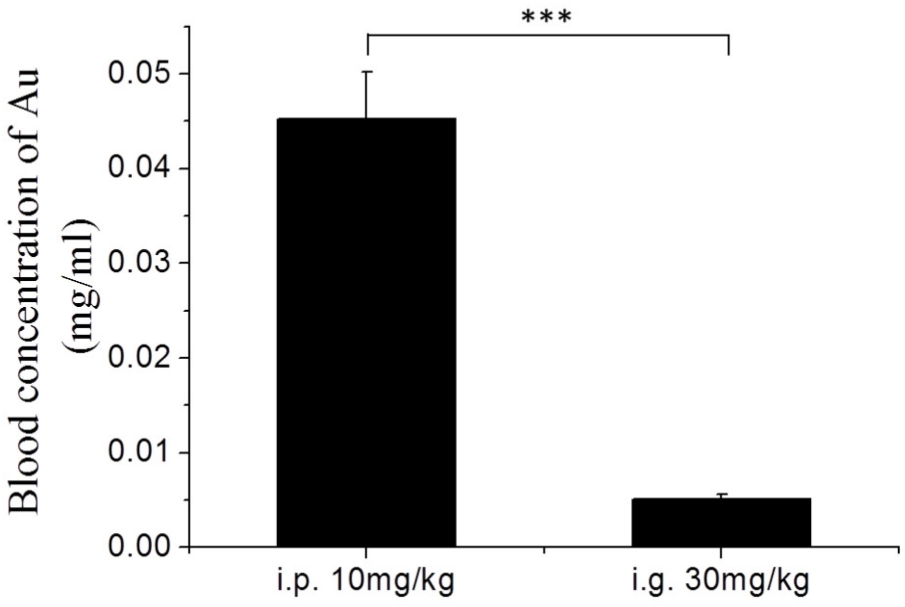

Figure S6. Comparison of blood concentration of Au after intraperitoneal injection (i.p.) and intragastric administration (i.g.) of Au clusters in rats for 10 days. Data are presented as the mean ± s.d. (n = 10); ***$P < 0.001$.

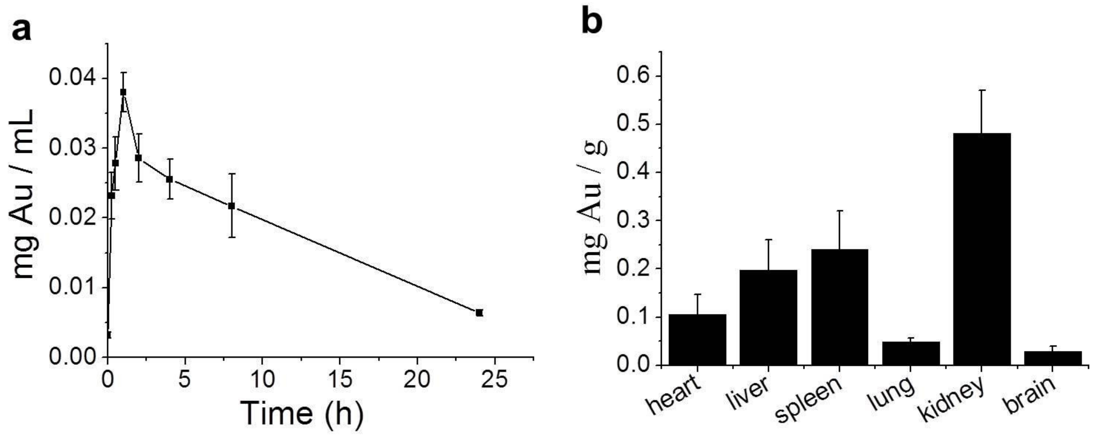

Figure S7. (a) Pharmacokinetic profiles of Au cluster after single intraperitoneal injection in rats. (b) Quantitative analysis of of Au distribution in various organs and tissues by ICP-MS after 6 weeks of Au clusters treatment. Data are presented as the mean ± s.d. (n = 3).

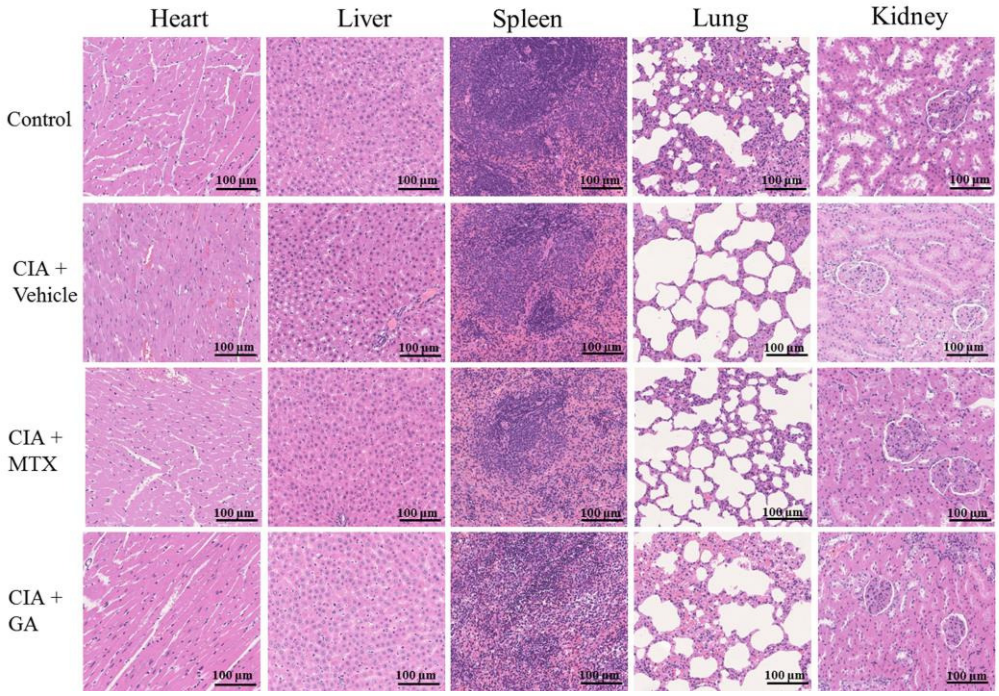

Figure S8. Representative histological analyses of major organ tissues of rats in non-immunized control rats and CIA rats treated with vehicle, MTX and Au clusters. Histological sections of the heart, liver, spleen, lung and kidney stained with hematoxylin and eosin. Data are representative of at least 6 rats (Scale bar = 100 μm.).

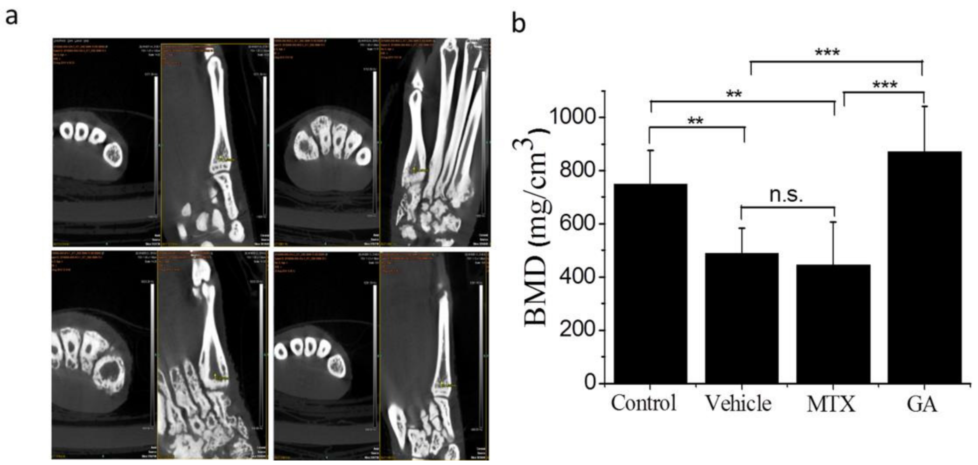

Figure S9. Treatment with Au clusters inhibits CIA-induced severe bone destruction. (a) Representative microCT coronal imaging of metatarsal bone articulations (n = 6) in CIA rats treated with vehicle, MTX and Au clusters on day 42. The microCT coronal imaging of metatarsal bone articulations in non-immunized rats was used as a control. The images are representative of six rats. (b) Bone mineral density (BDM) in all experimental groups. Data are presented as the mean ± s.d. (n = 6); $^{**}P < 0.01$, $^{***}P < 0.001$; n.s., not significant.

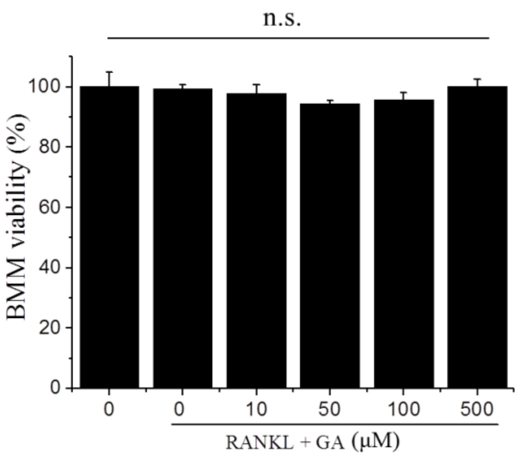

Figure S10. Viability of bone marrow-derived monocytes (BMMs) treated with different concentrations of Au clusters in the presence of 50 ng/ml RANKL for 24 h at 37 °C. Viability of BMMs in the absence of RANKL or Au clusters was used as a control. Data are presented as the mean ± s.d. (n = 8); n.s., not significant.

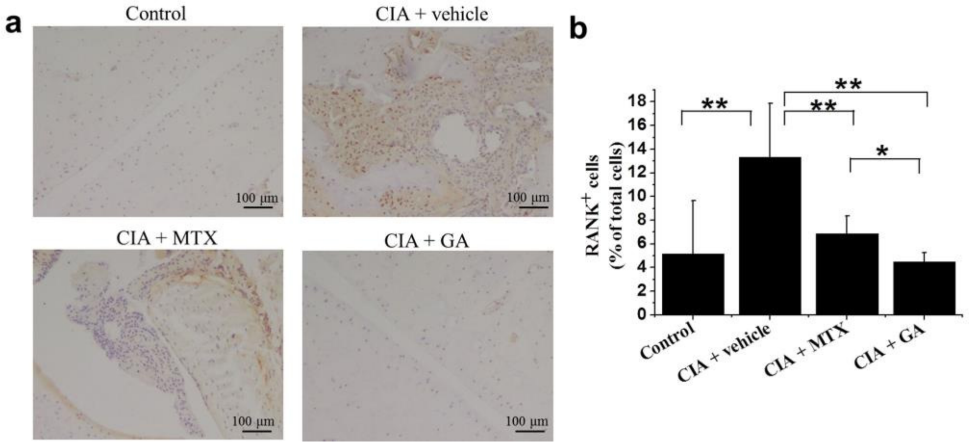

Figure S11. Representative images of immunohistochemistry of RANK antigenicity (a) (3 mice per group) and percentages of RANK+ cells (b) in joint tissues from non-immunized rats and CIA rats treated with vehicle, MTX and Au clusters on day 42. The RANK antigenicity in joint tissues of the Au cluster-treated CIA rats is similar to that of non-immunized rats (Scale bar = 100 μm.).

2. Supplementary Tables

Table S1. Complete blood counts of non-immunized rats (control) and CIA rats after vehicle, MTX and Au cluster treatment (n = 6).

	Control	CIA+vehicle	CIA+MTX	CIA+GA
WBC（10^9/L）	7.50±1.00	4.40±1.00$^{\#}$	9.70±1.00	10.30±4.50
RBC（10^{12}/L）	8.10±0.06	7.95±0.30	8.37±0.26	7.57±0.26
HGB（g/L）	152.33±1.15	152.33±9.86	155.33±6.50	135.33±3.51
HCT（%）	43.93±0.77	45.03±2.27	45.70±1.57	40.47±1.59
MCV（fL）	54.00±1.73	56.67±0.58	54.67±0.58	53.67±4.04
MCH（pg)	18.80±0.26	19.20±0.62	18.53±0.15	17.90±0.95
MCHC（g/L）	346.67±4.93	338.67±6.11	339.33±3.21	334.67±4.72
PLT（10^9/L）	760.67±157.90	258.33±151.7$^{\#}$	538.33±226.40	618.00±415.00

Abbreviations: WBC, white blood cell count; RBC, red blood cell count; HGB, haemoglobin; HCT, haematocrit; MCV, mean corpuscular volume; MCH, mean corpuscular haemoglobin; MCHC, mean corpuscular haemoglobin concentration; and PLT, platelet count (PLT). Data are presented as the mean ± s.d.; $^{\#}P < 0.05$ versus control.

Table S2. Blood biochemical indexes of non-immunized rats (control) and CIA rats after vehicle, MTX and Au cluster treatment (n = 6).

	Control	CIA+vehicle	CIA+MTX	CIA+GA
ALT (U/L)	30.70±3.05	26.67±3.79	26.33±0.58	22.27±2.00
AST (U/L)	134.67±26.08	121.67±14.15	110.67±9.07	81.95±8.71
TBIL (μmol/L)	1.38±0.08	1.54±0.24	1.17±0.11	1.40±0.34
BUN（mmol/L）	11.72±0.27	12.44±0.59	10.84±0.95	8.57±1.10
CREA (μmol/L)	24.38±0.65	25.81±1.29	21.44±1.33	18.89±2.21

Abbreviations: ALT, alanine aminotransferase; AST, aspartate aminotransferase; TBIL, total bilirubin; BUN, blood urea nitrogen; and CREA, creatinine. Data are presented as the mean ± s.d.

3. Supplementary Methods

Pathological score method**:** Pathological scores were assessed by grading the different joint tissues from 0 to 3 according to the degree of inflammatory injury: -/0, no abnormalities seen in the periarticular soft tissues, synovial tissues, cartilage or bone; +/1, mild edema in the local soft tissue around the joints, a small amount of neutrophil and lymphocyte infiltration, slight edema in the synovial tissue around the cartilage, swelling and monolayer arrangement of the synovial cells, slightly damaged articular cartilage tissue, pannus formation in the articular cartilage, basically complete articular surface, local visible individual trabecular bone fracture, marrow cavity fusion, and no inflammatory reaction in the bone marrow cavity; ++/2, obvious edema in the soft tissue around the joint, small vascular congestion, a large amount of

neutrophil and lymphocyte infiltration, local inflammatory granulation tissue formation, obvious edema in the synovial tissues around the cartilage, obvious swelling of synovial cells, hyperplasia and multi-layer alignment, scattered infiltration of neutrophils and lymphocytes around the synovial tissue, obviously damaged articular cartilage, local visible cartilage defects, inflammatory fibrous tissue hyperplasia at the defect site, obviously damaged bone tissue, local trabecular bone fracture, bone marrow cavity fusion, and neutrophil infiltration in the bone marrow cavity (or bone marrow cell hyperplasia), but no inflammatory tissue hyperplasia in the bone marrow cavity; and +++/3, significant edema in the soft tissues around the joints, small vascular congestion, a large amount of neutrophil and lymphocyte infiltration, local inflammatory granulation tissue hyperplasia, a large amount of granuloma and abscess formation, significant proliferation of synovial tissue around the cartilage, significant inflammatory reaction, capillary hyperplasia and inflammatory granuloma formation, accumulated inflammation throughout the articular cavity, severely damaged articular cartilage, inflammatory fibrous tissue replacing the defect site, bone tissue severely eroded by inflammatory tissue, inflammation in the bone marrow cavity, a great deal of inflammatory fibrous tissue hyperplasia in the marrow cavity, and bone marrow is replaced by inflammatory fibrous tissue.

Molecular modelling of $Au_{29}SG_{27}$: The spatial structure of $Au_{29}SG_{27}$ molecules was optimized by density functional theory (DFT) calculations. First, we used the "divide and protect" approach[33] to expand the $Au_{29}SG_{27}$ molecular formula into

possible gold cores complexed by different staple motifs. $Au_{29}SG_{27} = Au_{a+a'} + [Au(SG)_2]_b + [Au_2(SG)_3]_c + [Au_3(SG)_4]_d$..., where a/a' denotes the number of surficial/non-surficial atoms in the gold core; and b, c and d... represent the numbers of different staple motifs. These numbers satisfy the following equation group: $a + a' + b + 2c + 3d + \ldots = 29$ and $2b + 3c + 4d + \ldots = 27$ according to the $Au_{29}SG_{27}$ molecular formula. Moreover, because each staple motif bonds to two superficial gold atoms with terminal sulphur atoms in GSH, there is the relationship $a = 2(b + c + d + \ldots)$. Under the above constraints, we can achieve serial possible structural divisions as $Au_4[Au_{12}(SG)_{13}][Au_{13}(SG)_{14}]$, $Au_4[Au_{11}(SG)_{12}][Au_{14}(SG)_{15}]$, $Au_4[Au_{10}(SG)_{11}][Au_{15}(SG)_{16}]$, etc. In this study, $Au_4[Au_{12}(SG)_{13}][Au_{13}(SG)_{14}]$ was adopted for its highest point symmetry.[34] Second, we simplified the molecular structure into $Au_4[Au_{12}(SCH_3)_{13}][Au_{13}(SCH_3)_{14}]$ by substituting $–SCH_3$ groups for the –SG ligands for computational cost considerations.[35] Third, we optimized the spatial structure of $Au_4[Au_{12}(SCH_3)_{13}][Au_{13}(SCH_3)_{14}]$ using the Gaussian09 program. The calculations used the generalized gradient approximation (GGA) functional Perdew-Burke-Ernzerhof (PBE). The basis set for gold is LANL2DZ, and the basis set for carbon, hydrogen, sulphur, oxygen and nitrogen is 6-31G(d).[33b, 34c] The stable structure of $Au_4[Au_{12}(SCH_3)_{13}][Au_{13}(SCH_3)_{14}]$ was achieved through massive calculations. Finally, we changed the coating ligands back to $Au_4[Au_{12}(GSH)_{13}][Au_{13}(GSH)_{14}]$ and re-optimized its stable spatial structure as shown in Figure 1.

Pharmacokinetics and biodistribution analysis of Au clusters after

intraperitoneal injection: For pharmacokinetic analysis, rats were intraperitoneally injected with Au clusters (5 mg of Au/kg in 1 ml saline), and blood samples (100 μl) were collected from the jugular vein at predetermined time points. The blood content of Au clusters was analyzed with inductively coupled plasmamass spectrometry (ICP-MS, Thermo-X7).

For in vivo biodistribution studies of Au clusters after six weeks of treatment, the rats were euthanized by CO_2 exposure and heart, liver, spleen, lung, kidney and brain were collected. the tissues samples were lyophilized, weighed and predigested by H_2O_2 and HNO_3 (1:3, v/v) overnight. The sample was further digested using aqua regia by mild heating. The remaining 0.1-0.2 ml sample solution was diluted with 2% HNO_3 and 1% HCl to a final volume. The sample solution was analyzed for Au content using ICP-MS. The experiment was conducted in triplicate.

References

[33] a) H. Hakkinen, M. Walter, H. Gronbeck, J, Phys. Chem. B. **2006**, 110, 9927; b) Y. Pei, R. Pal, C. Y. Liu, Y. Gao, Z. H. Zhang, X. C. Zeng, J. Am. Chem. Soc. **2012**, 134, 3015.

[34] a) Y. B. Song, S. X. Wang, J. Zhang, X. Kang, S. Chen, P. Li, H. T. Sheng, M. Z. Zhu, J. Am. Chem. Soc. **2014**, 136, 2963; b) X. K. Wan, W. W. Xu, S. F. Yuan, Y. Gao, X. C. Zeng, Q. M. Wang, Angew. Chem. Int. Ed. Engl. **2015**,

54, 9683; c) H. Wang, X. Li, L. Gao, J. Zhai, R. Liu, X. Y. Gao, D. Q. Wang, L. Zhao, Nanoscale **2016**, 8, 11454.

[35] a) A. Das, T. Li, K. Nobusada, C. Zeng, N. L. Rosi, R. Jin, J. Am. Chem. Soc. **2013**, 135, 18264; b) C. Liu, S. Lin, Y. Pei, X. C. Zeng, J. Am. Chem. Soc. **2013**, 135, 18067.
